# Amylase Binding to Oral Streptococci: A Key Interaction for Human Oral Microbial Ecology, Adaptation and Fitness

**DOI:** 10.3390/biom15111616

**Published:** 2025-11-18

**Authors:** Amarpreet Sabharwal, Elaine M. Haase, Frank A. Scannapieco

**Affiliations:** 1 Schulich School of Medicine and Dentistry, Western University, London, ON N6A 5C1, Canada; amarpreet.sabharwal@schulich.uwo.ca; 2 Department of Oral Biology, School of Dental Medicine, University at Buffalo, Buffalo, NY 14214, USA; emhaase6570@gmail.com

**Keywords:** dental plaque, saliva, biofilm, bacterial colonization, microbial evolution

## Abstract

The interaction between human salivary alpha-amylase (HSAmy) and amylase-binding oral streptococci (ABS) helps determine the bacteria that colonize the oral cavity by establishing dental biofilms. Streptococci are important pioneer species of the oral cavity and influence oral health as well as common diseases such as dental caries. Various oral streptococcal species express distinct amylase-binding proteins, among which amylase-binding protein A (AbpA), encoded by the *abpA* gene in *Streptococcus gordonii* and several other species, which is the most extensively studied. Amylase binding facilitates microbial adhesion to host surfaces and biofilm formation and enables bacteria to harness the host’s amylase enzymatic activity at their cell surface, enhancing their capacity to metabolize dietary starch for nutritional gain. Additionally, amylase binding may also influence bacterial cell division and stress tolerance by engaging novel bacterial signaling pathways. From an evolutionary perspective, both Neanderthals and modern humans exhibit functional adaptations in nutrient metabolism, including selection for salivary amylase-binding oral streptococci, highlighting the importance of microbial co-adaptation in response to host diet. Further research is warranted to elucidate the broader roles of amylase binding to bacteria in host-bacterial signaling, bacterial cell division and fitness and the evolutionary trajectory of the oral microbiome.

## 1. Introduction

The human oral cavity represents a complex and dynamic ecosystem where the resident microbiome is constantly exposed to changing environmental conditions, including changes in pH, nutrients, salivary flow and composition and mechanical forces [1]. For effective colonization and persistence in the mouth, microbes have developed numerous strategies for adaptation to their environment. Commensal oral streptococci are pioneer colonizers of early dental biofilm on oral hard and soft tissues. Since dental biofilm formation is essential to the pathogenesis of dental caries and periodontal disease, understanding molecular mechanisms of host–microbe and microbe–microbe interactions may hope to inform future diagnostic and therapeutic approaches for these common disorders.

Saliva provides an important influence in oral microbial ecology [2]. One salivary component of particular significance in this regard is amylase, an abundant enzyme produced by serous cells of the parotid gland but also by sublingual, submaxillary and minor salivary glands. The amylase concentration in saliva varies between 0.04 and 0.4 mg/mL and may constitute up to 5% of the total salivary protein. Salivary stimulation can dramatically increase the levels of amylase. The enzyme’s main catalytic specificity is to hydrolyze the alpha-1,4-glucosidic bonds of starch, glycogen and other polysaccharides, releasing maltose and maltodextrin that provides an abundant source of carbohydrate for oral bacterial nutrition.

Other interesting functions have been ascribed to amylase. An interaction between human salivary alpha-amylase (HSAmy) and certain oral streptococci has been well-documented and may play an important role in regulating the colonization of these bacteria in the mouth [3]. These amylase-binding streptococci (ABS) are abundant in the oral cavity. The interaction of HSAmy and ABS is mediated by amylase-binding protein(s) (ABP) [3]. Once bound to the bacterial surface, amylase retains enzymatic activity to mediate starch hydrolysis to fermentable oligosaccharides that contribute to bacterial nutrition [4,5]. The binding of amylase to the cell surface is inhibited by starch and maltotriose, but not maltose, supporting the involvement of specific receptor(s) on the bacterial surface. The addition of amylase to culture medium containing starch enhances the growth of *Streptococcus gordonii* [6].

A critical aspect of the host–microbe interaction in the mouth is the ecological success of early tooth colonizers (particularly oral streptococci), which must first interact with the salivary pellicle, a thin layer of saliva on oral structures. Amylase-binding protein A (AbpA) has been identified and localized to the surface of the bacteria to promote the adhesion of amylase-binding streptococci to amylase-coated surfaces in vitro, which serve as an analog of the salivary pellicle. AbpA-deficient mutants of these streptococci produce less biofilm than parental strains under in vitro flow conditions [6]. The binding of HSAmy to bacteria is calcium-independent, suggesting a mechanism distinct from enzymatic hydrolysis. The active site of HSAmy mediates saccharide binding and the hydrolysis of starch, with multiple secondary oligosaccharide-binding sites thought to enhance amylase affinity to starch granules [7]. These secondary oligosaccharide-binding sites have also been shown to play a role in bacterial binding. Mutation of aromatic residues in the secondary oligosaccharide-binding sites decreased the affinity of HSAmy binding to *S. gordonii*. Because amylase bound to bacteria retains enzymatic activity [7,8], the bacterium-binding site is likely distinct from that involved in enzymatic activity. Mutations in aromatic residues of secondary oligosaccharide-binding sites did not reduce the binding of amylase to hydroxyapatite, suggesting other un-identified sites mediating this interaction [7].

The possibility that amylase binding to oral streptococci might influence dental caries has been addressed by several studies [9,10,11,12]. Interestingly, Amylase-binding protein B (AbpB), rather than AbpA, appears to be more important for colonization of teeth in rats eating a starch-rich diet, and its deletion was partially masked if rats consumed a sucrose-starch diet. *S. gordonii* was compared to *Streptococcus mutans* with respect to oral colonization of the teeth and cariogenicity in a well-characterized rat model. Mutants of *S. gordonii* deficient in glucosyltransferase (GtfG), amylase-binding proteins (AbpA/AbpB), and *S. mutans* glucosyltransferase (GtfB) were studied. While both *S. gordonii* and *S. mutans* were abundant colonizers of rat’s teeth in the presence of starch or sucrose diets, *S. mutans* always out-competed *S. gordonii* on the teeth. Caries induction reflects *S. mutans* or *S. gordonii* colonization abundance. *S. mutans* was found to be more cariogenic than *S. gordonii*. Thus, amylase binding to *S. gordonii* may not have a great influence on dental caries, especially when compared to the well-known cariogenic *S. mutans* exposed to a sucrose-rich diet.

The recent literature points towards a more complex role for the amylase-binding phenotype during the evolution of the human microbiome, where the emergence of ABS coincides with the introduction of plant starch in the human diet [13,14,15,16]. The goal of this review is to provide a summary of what is known to date of the relationship between HSAmy and ABS, and to provide suggestions for future studies.

## 2. The Genetic Basis and Diversity of ABPs

The ability of ABS to bind to HSAmy is not conferred by a single, conserved molecular mechanism. Instead, genetic and phylogenetic analyses suggest convergent evolution where several proteins, complemented by specialized processing systems, have adapted to perform a function that provides a selective advantage to the bacteria by interacting with an abundant host enzyme.

### 2.1. abpA-srtB Operon: System for Processing and Display in ABS

*S. gordonii*, a pioneer colonizer of tooth biofilm [17], expresses the protein AbpA [6], encoded by the *abpA* gene [18]. Genomic analysis has shown that it is part of a sophisticated expression and cell-wall anchoring system [19]. The *abpA* gene is co-transcribed with a proximally located downstream gene, *srtB*, that encodes a specialized class B sortase enzyme. The demonstration of co-transcription by PCR primers that span the gene junction confirms that *abpA-srtB* forms an operon for coordinated synthesis of the protein and the enzyme required for its cell wall localization [15]. Sortases are transpeptidases that covalently anchor surface proteins containing a conserved C-terminal sorting signal (e.g., LPXTG motif) to the peptidoglycan of Gram-positive bacteria [20]. While many bacteria possess a housekeeping sortase (like SrtA), which anchors a wide range of proteins, the AbpA-SrtB system in *S. gordonii* is distinct. The SrtB enzyme specifically recognizes a novel C-terminal cell-wall sorting motif within the AbpA protein [19]. The evolution of a dedicated sortase suggests a critical role for AbpA. A generic anchoring system may have been insufficient to ensure proper timing, density display, or conformation of AbpA on the cell surface, all of which are crucial for cell signaling roles. SrtB-mediated processing of AbpA can result in formation of a ladder profile in immunoblotting [19], suggesting that AbpA may undergo polymerization within the cell wall. The biological significance of this potential polymerization is not fully understood but seems specific for the processing of AbpA.

### 2.2. Multiple ABPs: Convergent Evolution and Horizontal Gene Transfer

Although the AbpA-SrtB system is well characterized in *S. gordonii*, it is not a universal mechanism for amylase binding among ABS. Extensive functional screening of diverse oral streptococcal species has shown a wide array of proteins that interact with amylase, varying in size from 20 to 87 kDa [21]. 

Phylogenetic and sequence analyses of ABPs have shown that they do not belong to a single, homologous protein family. ABPs cluster into at least six distinct and phylogenetically unrelated families: AbpA, AbpB and four novel families [21]. These novel protein families have been annotated based on homology to peptidoglycan-binding proteins, glutamine ABC transporters and choline-binding proteins. No single ancestral gene has been identified as an evolutionary source, suggesting convergent evolution by common environmental pressure from abundant salivary amylase in the oral cavity driving disparate genes towards a common functional solution. Further, comparative genomics of various streptococci provides evidence that acquisition of *abpA* was likely mediated by horizontal gene transfer [21].

## 3. Functional Characterization of ABPs

The functional significance of ABPs likely extends beyond roles in cell adhesion and nutrition. ABPs may also integrate environmental cues with cellular processes like cell division and stress tolerance by novel cell signaling pathways. Table 1 summarizes this evidence.

### 3.1. Canonical Functions: Adhesion and Nutrition

Following professional tooth cleaning, HSAmy adsorbs to hydroxyapatite to form part of the acquired enamel pellicle, creating a surface rich in receptors for ABS. AbpA has been shown in vitro to enhance the adhesion of *S. gordonii* to HSAmy-coated surfaces, a critical first step in early biofilm formation [6]. The AbpA-HSAmy interaction found to bind to amylase retains approximately 60% of its hydrolytic activity, allowing it to efficiently break down dietary starch into smaller, fermentable oligosaccharides like maltose and maltotriose directly at the cell surface [4,5,22]. The critical nature of this function is highlighted by the observation that preincubation of AbpA-deficient *S. gordonii* cells with salivary amylase, followed by washing in phosphate-buffered saline to remove unbound amylase, were unable to grow in a defined medium where starch was the sole carbohydrate source [6]. AbpB, annotated as a dipeptidase, also binds amylase, but it is not essential for amylase to bind to the cell surface of *S. gordonii* [23].

The roles of AbpA and AbpB in microbial ecology are complex (Table 1). While numerous in vitro studies demonstrate a role for AbpA in adhesion and biofilm formation, some in vivo studies show contradictory results. For example, a specific pathogen-free rat model of tooth colonization suggested that the expression of AbpA may, under certain conditions, inhibit colonization by *S. gordonii* [10]. Interestingly, when rats were inoculated with an AbpB mutant strain having intact AbpA, *S. gordonii* failed to colonize the teeth of starch-eating rats with abundant amylase in their saliva, but some colonization was restored with a starch/sucrose diet. Strains defective in AbpA colonized better than wild-type strains. AbpA appeared to inhibit colonization of the plaque biofilm in vivo. It was speculated that amylase/ABPs interact with glucosyltransferase or other colonization factors of these cells. These discrepancies suggest that the function of amylase binding by oral bacteria may be context dependent, with differences between initial adhesion to the host versus later stages of biofilm maturation and dispersal and further influenced by differences in host species.

### 3.2. Potential Roles for AbpA Beyond Adhesion and Nutrition

Microarray analyses examined the possibility that HSAmy binding to streptococci may affect genes involved in bacterial fitness [24]. Gene expression profiling showed that exposure to HSAmy elicits differential gene expression in wild-type *S. gordonii*, which is otherwise absent in an *abpA^-^* mutant. Genes involved in fatty acid synthesis were upregulated and associated with increased bacterial growth, pH resistance and resistance to triclosan, all phenotypic changes enhancing bacterial survival in the oral environment.

To identify proteins that may interact with AbpA to affect differential gene expression, phage-display experiments were undertaken to screen for peptides that bind directly to AbpA (unpublished data, Table 2; see information in Supplementary Methods). The phage display was performed using a Ph.D-7 kit following the manufacturer’s instructions. This unbiased approach showed potential interactions with some fatty acid-synthesis proteins previously identified, beta-ketoacyl-ACP reductase (FabG) [16], as well as with core components of the bacterial divisome [25], for example cell division protein FtsZ, an essential tubulin homolog that forms the foundational Z-ring supporting cell division [26] and penicillin-binding protein PBP2b, known to affect chain length in *S. pneumoniae* [27].

**Figure 1:**
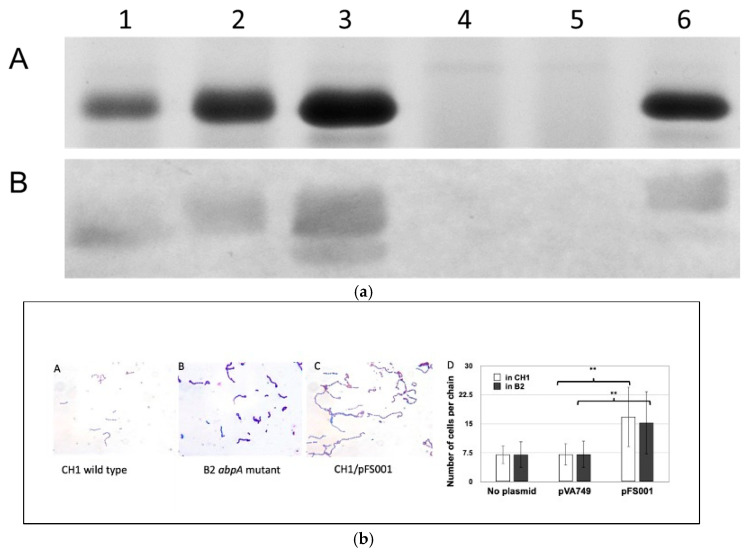
(**a**) Filter-concentrated supernatants from overnight TSBY cultures. Lane 1, CH1 (*S. gordonii* wild-type, parental strain); 2, CH1/pVA749 (parental strain with replicative plasmid); 3, CH1/pFS001 (parental strain with plasmid containing *abpA*); 4, B2 (AbpA-deficient CH1); 5, B2/pVA749 (AbpA-deficient CH1 with replicative plasmid); 6, B2/pFS001 (AbpA-deficient CH1 with plasmid containing *abpA*). A. Gel: Equal concentrations of protein were loaded onto 12% SDS-PAGE gel stained with AcquaStain (Bulldog Bio, Portsmouth, NH, USA). The major band of 20 kDa is AbpA. B. Far Western Blot: Proteins were transferred onto a PVDF membrane (Millipore-Sigma, Burlington, MA, USA), blocked with 3% skim milk in Tris-buffered saline with 0.1% Tween 20, incubated with purified human non-glycosylated salivary alpha-amylase, followed by primary antibody (rabbit anti-human amylase) and secondary antibody (goat anti-rabbit IgG conjugated with alkaline phosphatase (BioRad). Color was developed with nitro blue tetrazolium (NBT; Millipore-Sigma, Burlington, MA, USA). (**b**) Chain length of cells in mid-log phase. A, B, C, Gram stain. D, number of cells per chain. Strains: CH1 (wild type), B2 (*abpA^-^* mutant), B2/pFS001 (complemented mutant). Vectors: pVA749 (empty vector); pFS001 (pVA749 with *abpA*). ** Statistically significant (*p* < 0.05).

That interactions between AbpA and the bacterial cell divisome proteins are possible is suggested by previous electron microscopic studies that showed localization of HSAmy to the cell division septum in dividing streptococci [28]. Additional studies were conducted of overnight cultures entering exponential phase of *S. gordonii* wild-type and mutant strains. After crystal violet staining, the bacteria were observed under a microscope with 1000 magnification. Bacterial chain lengths were determined by counting the cells in 10 random streptococcal chains per field (10 fields for each strain). The average cell number of 100 chains was calculated and subjected to statistical analysis. When AbpA is over expressed by a multicopy plasmid, not only is more amylase bound, but the average chain length of *S. gordonii* cells is significantly lengthened in both wild-type *S. gordonii* CH1 and in an isogenic *abpA^-^* mutant strain cultured in vitro (unpublished data, Figure 1a,b; see information in Supplementary Methods). This co-localization suggests that AbpA may be a transient member of the ABS divisome, potentially serving to link environmental sensing directly to bacterial cell metabolic regulation.

### 3.3. AbpA-CTM Axis: Pathway for Oxidative Stress Resistance

Comparative transcriptomic analysis of wild-type *S. gordonii* and an isogenic *abpA^-^* mutant showed that a specific gene cluster (*ccdA1*/*tlpA*/*msrB*), homologous to the CTM gene cluster *(ccdA1*/*etrx1*/*msrAB2*) in *S. pneumoniae*, was consistently and significantly downregulated in the *abpA^-^* mutant across all growth conditions, even in the absence of HSAmy [15]. This constitutive, HSAmy-independent effect demonstrates that AbpA may play an important role in regulating this gene cluster, beyond the response to its ligand.

The CTM gene cluster in *S. pneumoniae* is involved in redox homeostasis and stress response [29,30]. The core components of this CTM homologous system in *S. gordonii* encode a cytochrome c-type biogenesis protein (CcdA1, a thioredoxin-like protein TlpA/etrx1 homolog) and a peptide methionine sulfoxide reductase (MsrAB homolog). The function of these components indicates a role in the management of oxidative stress. Methionine sulfoxide reductases (MsrA and MsrB) are important for ABSs that lack catalase [31]. These enzymes repair oxidatively damaged proteins by reducing methionine sulfoxide to methionine [32]. The functional outcome of this oxidative stress protection was tested on an *abpA^-^* mutant of *S. gordonii*, which was found to be significantly more sensitive to killing by extracellularly applied hydrogen peroxide when compared to an *abpA*-complemented strain [15]. Importantly, the mutant did not show an increased sensitivity to oxidative stress generated internally (by paraquat treatment) or to the oxidative burst within phagocytic cells (unpublished data; see information in Supplementary Methods). This distinction is important as it indicates that the AbpA-regulated CTM system may have evolved to defend against external oxidative injuries such as hydrogen peroxide produced by competing bacteria in oral biofilm.

The CTM gene cluster in *S. gordonii* and *S. pneumoniae* is flanked at the C-terminus sequence coding for a sensor histidine kinase and a response regulator. The histidine kinase and response regulator are homologous to the two-component system (TCS) in *S. pneumoniae* that is thought to be associated with a response to environmental stress [33] and multiple TCSs have been shown to regulate responses to environmental stresses in *S. gordonii* [34]. PCR studies in *S. gordonii* using intergenic primers suggest that the CTM, sensor histidine kinase and cognate response regulator genes may be transcribed as a single, long polycistronic message. The expression of this putative operon is influenced by AbpA as all genes in this cluster show some level of downregulation in the *abpA^-^* mutant. Interestingly, this downregulation occurs in a gradient, with the most upstream gene (*ccdA1*) showing the greatest decrease in expression and the most downstream gene (sensor histidine kinase) showing the least. This suggests a complex regulatory architecture possibly involving transcriptional polarity or attenuation within the operon. Phage display experiments raised the possibility of a potential physical interaction between AbpA and the sensor histidine kinase in this TCS. A direct signaling pathway in which AbpA (after conformational change or a cell surface interaction) directly modulates the activity of the sensor histidine kinase which then phosphorylates the response regulator to control transcription of the entire CTM-TCS locus is possible.

The role of an AbpA-CTM axis for defense against extracellular oxidative stress should be qualified against its ecological purpose. Pioneer colonizers like *S. gordonii* produce hydrogen peroxide via the *spxB*-encoded pyruvate oxidase to inhibit growth of other species in competition such as the cariogenic pathogen, *S. mutans* [35]. Constitutive upregulation by AbpA may allow ABS to withstand the oxidative conditions they generate to establish and defend themselves in the oral biofilm. In this regard, CTM may not be viewed as a generic stress-response system but as a defensive component to complement an active strategy by pioneer colonizers to establish superiority early in an ecological system.

## 4. Ecological and Evolutionary Considerations

Molecular mechanisms governing the binding of HSAmy to ABS are the result of a long and dynamic co-evolutionary history with their mammalian hosts, shaped by dietary shifts, ecological competition and constant pressure to adapt and survive in a complex oral biofilm.

### 4.1. Co-Evolution: Interactions of Diet, Genes and Microbiome

This host–microbe co-adaptation is not a recent event. Analysis of dental calculus from ancient hominids, including Neanderthals and Late Pleistocene modern humans, revealed the presence of substantial proportions of amylase-binding oral streptococci [13,14]. This indicates that the selective pressures for HSAmy-ABS like interactions predate agriculture by tens of thousands of years, likely linked to earlier dietary shifts that incorporated starch-rich foods such as tubers into the hominid diet. A prominent event in human evolution was a significant increase in starch consumption, particularly following the Neolithic revolution and the advent of agriculture [36]. This dietary shift likely created a strong selective pressure for the extraction of energy from starch-rich foods and is reflected by the expansion of AMY1 gene copy numbers [37]. Populations with a history of high-starch diets tend to have more AMY1 copies and consequently higher concentrations of amylase in their saliva [36,38]. This change in host physiology likely had a persistent effect on the ecology of the oral microbiome [39]. The increased availability of HSAmy and its starch-derived byproducts created a new and nutrient-rich niche. Oral streptococci that evolved and acquired the ability to bind to HSAmy enjoyed a substantial fitness advantage.

### 4.2. Amylase-Binding Bacteria in Other Mammals

The HSAmy-to-ABS binding phenotype is not restricted to the human oral microbiome. A comparative study of dental biofilms in 14 non-human mammals showed ABS ranging from 2 to 31% of total microbiota in five out of six amylase-secreting animals [40]. Pigs, which secrete amylase, were a notable exception. All animals that did not secrete amylase in their saliva did not have ABS. *Streptococcus suis* (*S. suis*) is an important colonizer of pig tonsils and must adhere to host cells for persistence and infection [41]. Adhesion proteins streptococcal adhesin P (SadP) and amylopullulanase A (ApuA) are both processed by SrtA for cell wall anchoring [42], and ApuA has alpha-amylase activity similar to AbpA [43]. A SrtA knockout can reduce adhesion in S. suis [44]. While AbpA is processed and anchored to the cell wall by SrtB in ABS and there is lack of evidence of a homologous AbpA/SrtB system in *S. suis*, it is likely that the carbohydrate-based adhesion in *S. suis* is the predominant mechanism of adhesion [42]. A combination of gene functions in distinct but related bacteria can account for oral microbiome adaptation in diverse hosts with similar ecological demands, i.e., oral cavities in humans and animals.

## 5. Future Directions

The major amylase-binding protein, AbpA, is co-transcribed with a proximally encoded novel sortase B. The potential multifunctionality of AbpA suggests it to be a novel protein by which bacteria may sense carbon sources and oxidative stress and react to a rapidly changing environment. In addition, the interaction of amylase with oral streptococci offers a unique model for host–protein interactions with commensal microflora. Fluctuations in amylase concentration in saliva could modulate the numbers of these organisms in plaque, in part through the mechanisms described above, with consequent effects on oral disease susceptibility. Future studies should further explore the HSAmy-AbpA interaction that may impact bacterial fitness to devise innovative approaches to control oral biofilms. The role of AbpA as a potential member of the streptococcal divisome also deserves attention. Additional studies are required to understand the role of amylase, ABS and starch in oral diseases such as dental caries and periodontal disease. Finally, the finding that the emergence of ABS as prominent members of the oral microbiome coincides with the adoption of starch in the human diet emphasizes the importance of this interaction in the evolutionary context and deserves further study.

It may be possible to design analogs that serve as inhibitors or promoters of microbial colonization and/or pathogenicity, with possible impact on disease prevention or control. This knowledge could extend beyond oral biofilm formation with potential application to other microbial communities affecting systemic health and disease.

**Figure 2:**
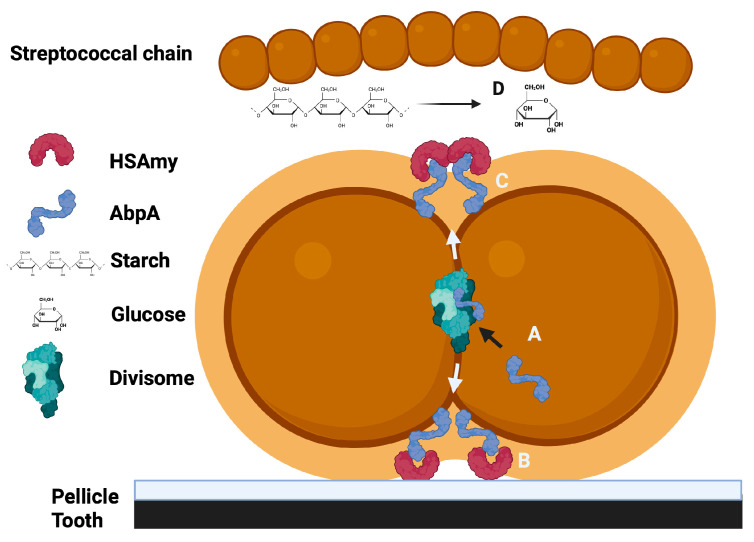
A summary of the potential roles for the HSAmy-AbpA interaction. A. AbpA is expressed, incorporated into the cell divisome and then released to the cell surface at the cell division septum. B. AbpA binds to HSAmy within salivary pellicle to promote bacterial adhesion to the tooth. C. HSAmy binds soluble enzymatically active HSAmy. D. Dietary starch is hydrolyzed, releasing simple sugars that can be utilized by the bacteria for energy.

In summary, the binding of HSAmy to AbpA appears to have multiple implications for the adhesion of bacteria to the host, the metabolism of dietary starch for bacterial nutrition and impacts cell division and signaling (Figure 2).

## 6. Conclusions

The interaction of amylase on the surface of common and abundant oral streptococcal species likely confers an important adaptation impacting the fitness of these organisms within the host. A sophisticated system appears to have evolved to bind amylase to the surface of oral streptococci and possibly mediate signals from the host to the bacteria that influence cell division, growth, adhesion and colonization. The bacterial amylase-binding phenotype has been an important ecological determinant during the evolution of the oral microbiome. Further studies are required to determine the potential role of amylase binding in bacterial cell-signaling, cell division, overall bacterial fitness, oral disease pathogenesis and the evolution of the oral microbiome.

## Figures and Tables

**Table 1:** Summary of evidence of amylase-binding proteins (ABPs) in oral ecology.

ABP families	
ABP family	Features
AbpA	~20 kDa; amylase-binding adhesin; SrtB anchored; novel sorting motif
AbpB	~87 kDa, dipeptidase; binds amylase but not essential for surface binding
Other	~20 to 87 kDa; homologous to peptidoglycan-binding proteins, glutamine ABC transporters and choline-binding proteins
AbpA-SrtB operon and processing	
Component	
*abpA*	Co-transcription with *srtB*
*srtB*	Dedicated sortase that anchors AbpA via novel motif; distinct from SrtA
Functional roles of ABPs	
Function	
Adhesion	AbpA enhances adhesion to HSAmy-coated surfaces
Nutrition	Starch is converted to maltose and maltotriose and AbpA^-^ mutants cannot grow on starch only
Regulation	Amylase triggers gene expression changes via AbpA; increase in fatty acid synthesis and stress tolerance
Cell division	Putative divisome links (FtsZ, PBP2b); septal localization; microscopic observation of AbpA at septum and chain length change in AbpA^-^ mutants
AbpA and oxidative stress	
Gene	
ccdA1	Redox homeostasis; downregulated in AbpA^-^ mutants
tlpA (etrx1)	Thioredoxin-like; downregulated in AbpA^-^ mutants
msrB (msrAB)	Repairs methionine sulfoxide; AbpA^-^ mutants sensitive to external H_2_O_2_
Histidine kinase/response regulator	Sense and regulate; operon-like; gradient downregulation
Ecological and evolutionary aspects	
Evidence	Observation and implication
AMY1 copy number	High-starch groups have increased AMY1 and salivary amylase; selects for ABS niche
Ancient human calculus	ABS present in Neanderthals/Late Pleistocene humans and predates agriculture
Non-human mammals	ABS abundant in several amylase-secretors; absent otherwise; physiologic adaptation of microbiome
*S. suis* analogs	SadP/ApuA (SrtA) with alpha-amylase activity; convergence without AbpA/SrtB

**Table 2:** Phage display sequences that potentially bind AbpA.

Sequence	Protein BLAST (2.7.1)	Max Score	Matched Sequence	SGO#	Recurrence
TSNNNLL	cell division protein (FtsZ)	21.8	SNNNLL	SGO_0675	(1/64 phages)
IVTQIPM	beta-ketoacyl-ACP reductase (FabG)	18	QIPM	SGO_1693	(1/64 phages)
TGSTRPW	beta-ketoacyl-ACP reductase (FabG)	17.6	TGSTR	SGO_1693	(4/64 phages)
EKKNMMN	sensor histidine kinase	16.8	+MMN	SGO_1174	(3/64 phages)
SSHSVQR	penicillin-binding protein 2B (PBP2b)	16.3	SHSVQ+R	SGO_1449	(20/64 phages)

## Data Availability

The original contributions presented in this study are included in the article/Supplementary Material. Further inquiries can be directed to the corresponding authors.

## Supplementary Materials

The following supporting information can be downloaded at: https://www.mdpi.com/article/10.3390/biom15111616/s1, Methodology; original blot. Refs. [21,45,46,47,48,49,50,51] are cited in Supplementary Materials.

## Abbreviations

The following abbreviations are used in this manuscript:

ABS	Amylase-Binding Streptococci
HSAmy	Human Alpha-amylase
AbpA	Amylase-binding Protein A
AbpB	Amylase-binding Protein B
