# Supplementary material for: Amylase Binding to Oral Streptococci: A Key Interaction for Human Oral Microbial Ecology, Adaptation and Fitness
Source: Biomolecules. 2025 Nov 18;15(11):1616. doi: 10.3390/biom15111616 (PMC12650345; doi:10.3390/biom15111616)
Supplement: Supplementary file 1 [file biomolecules-15-01616-s001.zip › biomolecules-3907213-Original Western blot-resub.pdf]

Protein expression and amylase-ligand overlay assay

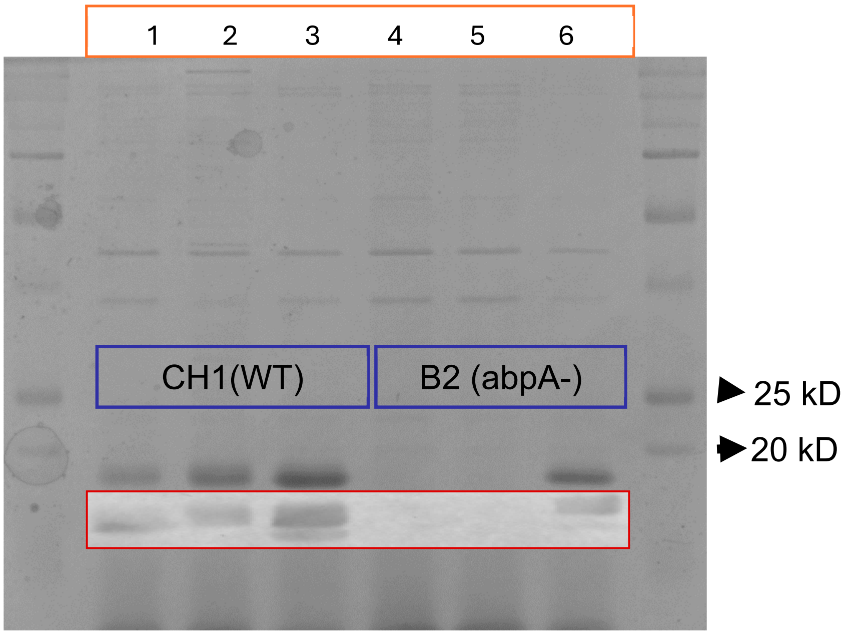

Filter-concentrated supernatants from overnight TSBY cultures. AquaBlue-stained protein gel with equivalent protein load per lane. 1, CH1; 2, CH1/pVA749; 3, CH1/pVA749:abpA; 4, B2; 5, B2/pVA749; 6, B2/pVA749:abpA.
Inset: Binding of amylase to the 20-kD protein (AbpA).
