# Supplementary material for: Amylase Binding to Oral Streptococci: A Key Interaction for Human Oral Microbial Ecology, Adaptation and Fitness
Source: Biomolecules. 2025 Nov 18;15(11):1616. doi: 10.3390/biom15111616 (PMC12650345; doi:10.3390/biom15111616)
Supplement: Supplementary file 1 [file biomolecules-15-01616-s001.zip › biomolecules-3907213-supplement-resub.pdf]

Supplemental Information of Methods

Screening of proteins interacting with AbpA by phage display.

The phage display experiments performed were performed using Ph.D07. Phage display libraries following manufacturer's instructions (NEB #E8100S). Briefly, 80 µg/ml AbpA (1.5 mg/mL) was incubated overnight on the surface of a petri dish (100 mm?) at 4°C and blocked with 5 mg/mL BSA. The phage library was diluted with 1 mL TBST (Tris buffered saline with 0.1% Tween 20), applied to the petri dish and incubated at room temperature for 1 h. The plate was washed with TBST ten times and the remaining phage on the petri dish was eluted with 0.1 M Glycine-HCL (pH 2.2) containing 1 mg/mL BSA. the eluate was then neutralized with 1M Tris-HCL (pH 9.1). A small amount of eluate was used for titering and the remainder amplified in *E. coli*. The above steps (panning) were repeated with amplified phage from the first panning instead using TBST (TBS with 0.5% Tween 20) as the washing buffer. After the third panning, the remaining phages were used to infect *E. coli* and were incubated with *E. coli* on a LB/IPTG/X-Gal agar plates. Single phage plaques were picked, amplified, and sequenced. Two independent phage display experiments were performed. Ph.D-12 phage display experiments were also performed in the same way.

Confirmation of mutation and complementation by Far-Western blotting.

Far-Western blot analysis was performed as previously described for the amylase-ligand binding (21). Briefly, culture supernatants were collected by centrifugation from 10 ml overnight cultures, followed by concentration and desalting with a centrifugal filter (Amicon® Ultra-4 Centrifugal Filter Unit, Millipore). The protein concentration of the concentrated supernatants was determined using the Pierce™ BCA Protein Assay Kit (Thermo Scientific). Supernatant proteins were resolved by gel electrophoresis on a 12% SDS-PAGE gel (20 µg protein/lane). Proteins in the gel were stained by AcquaStain (Bulldog Bio) or transferred onto polyvinylidene difluoride (PVDF) membrane (Millipore). The PVDF membrane was blocked with 3% skim milk in Tris buffered saline with 0.1% Tween 20 (TBST), then incubated sequentially with purified human non-glycosylated salivary α-amylase (0.1 mg/ml), primary antibody (rabbit anti-human amylase; and secondary antibody (goat anti-rabbit IgG conjugated with alkaline phosphatase; Bio-Rad). Color was developed with substrate nitroblue tetrazolium (NBT; Sigma). Purified salivary amylase (10 ng) was added as a positive control in the same blot and confirmed the quality of the reagents (data not shown).

Determination of Chain length

S. gordonii strains (Table S1) were routinely cultured from frozen stocks to tryptic soy broth with 0.5% yeast extract and 1.5% Bacto agar (TSBY; Becton Dickinson [BD]) and incubated for 48 h at 37°C in a candle jar, unless otherwise indicated. For some experiments, strains were cultured in Todd-Hewitt (TH;BD) medium or chemically defined medium (DM) containing sugars as indicated (45,46). Media were supplemented as needed with the appropriate antibiotics (250 µg/ml spectinomycin and/or 5 µg/ml erythromycin).

Mutant construction and complementation.

The AbpA-deficient *S. gordonii* strain CH0944 was constructed by allelic replacement of a 361-bp internal fragment of the *abpA* structural gene with a promoter-less, terminator-less aad9 spectinomycin resistance gene. This construct retains the regulatory cabolite responsive element (CRE) located 153-bp downstream of the translation start site (47) and allows read through of the co-transcribed downstream sortase B (*srtB*) gene.

To construct the mutant strain, DNA fragments corresponding to the chromosomal regions flanking the desired replacement region in parental strain CH1 were PCR-amplified from chromosomal DNA template using primers listed in Table S2, cloned into pBN2010 carrying aad9, and subsequently transformed into chemically competent *E. coli* CopyCutter cells (Epicentre). Putative transformants were screened on LB agar plates containing spectinomycin (50 µg/ml), and the presence of plasmid was confirmed by colony PCR. The 1.6-kb fragment carrying aad9 flanked by the desired chromosomal regions was amplified from transformant DNA by primers *abpA*-UF and *abpA*-DR. After confirming the amplicon sequence fidelity, the linear double-stranded DNA fragment was transformed into serum-competent *S. gordonii* CH1 cells. (48). TH plates with 250 µg/ml spectinomycin were used to select transformants. The fidelity of the desired chromosomal mutation was confirmed by sequencing an amplicon generated by PCR using primers *abpA*-KF and *abpA*-KR with genomic DNA from strain CH0944 as template.

To complement strain CH0944, the streptococcal replicative plasmid pFS001containing a copy of *abpA* (Haase et al., 2015) was transformed into serum-competent *S. gordonii* CH0944 cells. Transformants were selected on TH plates containing spectinomycin and erthromycin.

CH1 (WT), B2 ($abpA^{-}$ mutant). Streptococcal strains containing multicopy plasmid pVA749:*abpA* (pFS001) result in increased levels of AbpA, in both WT or complemented mutant. Strains expressing these high levels of AbpA exhibited increased chain length compared to strains without vector or with empty vector (pVA749) suggesting that AbpA interferes with the late steps of cell division and interacts with and/or influences the functions of some cell division proteins.

Sensitivity to paraquat. Paraquat (Sigma) was used to generate intracellular oxidative stress according to the method of Jakubovics et al. (49). Briefly, overnight cultures of CH1 and CH0944 grown in TSBY with antibiotics, as appropriate, were subcultured to DM-0.8% glucose and grown to mid-log phase. Cells from 5 ml of each strain were pelleted by centrifugation and suspended in 5 ml of PBS (pH 7.5). Paraquat was added to 10 mM into a 200-µl aliquot of cell suspension and incubated for different times (0, 15, 30, 60 min). The viability of paraquat-treated bacteria was quantified by CFU. The relative viability was calculated as the ratio in CFU/ml of the treated/non-treated samples. Three independent experiments were performed with six technical replicates per experiment. Mean CFUs were calculated and Student's *t*-test was applied to estimate statistical significance.

Effect of AbpA on resistance to phagocytosis. Bacterial resistance to phagosomal killing was assessed given that the phagosome is generally highly dependent on generated oxidative stress to kill bacteria (50). Both the rate of bacterial uptake (phagocytosis) by the macrophage and bacterial survival within the macrophage were quantified.

To quantify bacterial uptake, biotin-labeled bacteria were prepared by washing mid-log phase bacteria in PBS, re-suspending in 0.5 ml 10 mM $NaIO_4$ in 1 M acetate (pH 5.5), and incubating on ice for 30 min. The cells were then washed and resuspended in 500 µl PBS with Biotin-XX-hydrazide (0.1 mg/ml) and Alexa 488 Hydrazide (0.01 mg/ml) (Life Technologies) and incubated at room temperature for 1 h. The OD_{600} was determined to estimate the number of bacteria. All bacteria were sonicated (Branson sonicator with microtip, setting 7) for 30 s to break the streptococcal chains before adding to the macrophage culture.

To determine the ability of bacteria to survive within the macrophage, a gentamicin protection assay was performed (51). To prepare macrophages for phagocytosis, confluent macrophage cultures were grown in complete medium (RPMI 1640 (Corning Cellgro) supplemented with 10% fetal bovine serum (Caisson) and 2 mM L-glutamine (Corning) and distributed into 12-well plates (1 ml/well), with six

wells containing microscope coverslips (Azer Scientific). After overnight incubation at 37°C in 5% CO_2, the macrophages were activated with 100 ng/ml LPS (*Salmonella enterica*, Sigma-Aldrich) and incubated for 1 h under the same conditions. Macrophages were enumerated in a counting chamber. The bacterial strains at mid-log phase, labeled as above, were added into the induced macrophages at a MOI of 10:1.

The plates containing bacteria and macrophages were centrifuged at 183 g for 1 min to enhance cell-to-cell contact, followed by incubation in 5% CO_2 at 37°C for 30 min. After incubation, each microscope coverslip was washed once with cold PBS and incubated with Alexa Fluor 594 streptavidin (Life Technologies) at 4°C for 5 min. The coverslips were washed with PBS and incubated with 4% paraformaldehyde (Electron Microscopy Sciences) for 30 min at room temperature to fix the cells. Each coverslip was mounted onto a glass slide and observed under an epi-fluorescence microscope (TE2000-U, Nikon). Differential staining of bacteria was used to indicate the location of the bacteria relative to the macrophage; internalized bacteria were stained green with Alexa Fluor 488, while biotinylated bacteria external to the macrophages were stained both green with Alexa Fluor 488 and red with Alexa Fluor 594 streptavidin. To quantify bacteria taken up into the macrophage, green-only colored bacteria were quantified from at least 50 macrophages. The phagocytic index was calculated as the average number of bacteria in each macrophage after 30 min of phagocytosis.

To quantify the surviving internalized bacteria, in the wells without coverslips, after 30 min incubation the medium was supplemented with 300 μg/ml gentamicin (Amresco) and 100 μg/ml ampicillin (Sigma) to kill bacteria outside the macrophage and incubated for another 2 h. The macrophages were washed with PBS and then lysed with 1 ml sterile water. Surviving bacteria released from within the macrophages were serially diluted and quantified by colony-forming units. The survival rate of bacteria within the macrophages was determined using the equation: Survival rate = ((Surviving bacteria per well) / (average of uptake bacteria per macrophage x macrophages per well) x 100%. These data were from three parallel samples obtained from each of three independent experiments performed on different days.

Table S1 Bacterial strains and plasmids relevant to unpublished parts of research covered in this review

Strain, plasmid, primer	Relevant Characteristics
S. gordonii Challis CH1	Parental strain
CH1/pVA749	Parental strain containing replicative plasmid
CH1/pFS0001	Parental strain containing replicative plasmid with abpA
CH0944	Internal fragment 361-bp of abpA replaced by promoter-less, terminator-less aad9, AbpA-deficient
CH0944/pVA749	AbpA-deficient mutant containing replicative plasmid
E. coli DH5α*	Cloning host
E. coli CopyCutter EPI400*	Cloning host
pBN2010	pBS SK+ carrying promoter-less, terminator-less aad9, SpR, ApR
pBN2010:abpA-Down	pBN2010 carrying a 423-bp region downstream of abpA, SpR, ApR
pBN2010:abpA-Down-UP	pBN2010:abpA-Down carrying a 384-bp upstream region of abpA, SpR, ApR
pVA749	Streptococcal replicative plasmid, EmR
pFS001	pVA749 carrying a 1005-bp insert abpA and upstream promoter region, EmR

**E. coli* DH5α was obtained from Stratagene and *E. coli* CopyCutter EPI400 was obtained from Epicentre

Table S2 PCR primers relevant to unpublished parts of research covered in this review

Primer name	Relevant Characteristics
abpA-UF	5′-ATCTCGAGTGTTACAATATTACACATTTCTG-3′; XhoI, deletion of *abpA*
abpA-UR	5′-TTAAGCTTAACCATCATGTTGAGCCAAGTATTC-3′; HindIII, deletion of abpA
abpA-DF	5′-TAGAATTCAGGTAAAGCTGCTGGTAAAGC-3′; EcoRI, deletion of *abpA*
abpA-DR	5′-ATGGATCCAGCATAAACATAAGCAATAGCTTC-3′; BamHI, deletion of *abpA*
abpA-KF	5′-AGGAACCATCTGTCACGCAG-3′
abpA-KR	5′-AAGTCCATTTCCCTTGCCGT-3′
aad9-F	5′-CCGTGGAATCATCCTCCCAAA-3′
aad9-R	5′-GATTCAGCCACTGCATTTCCC-3′
pVA749-F	5′-CTCGCGCTCTAAACGCTCTA-3′
pVA749-R	5′-GGCAGTTGAAAGTCAGCACC-3′
srtB-F	5′-GAATGAGCCTTACATGGGGA-3′
srtB-R	5′-TACTTCCACGAGCATGACCA-3′
